# Mendelian Randomization Analysis Identifies CpG Sites as Putative Mediators for Genetic Influences on Cardiovascular Disease Risk

**DOI:** 10.1016/j.ajhg.2017.09.003

**Published:** 2017-10-05

**Authors:** Tom G. Richardson, Jie Zheng, George Davey Smith, Nicholas J. Timpson, Tom R. Gaunt, Caroline L. Relton, Gibran Hemani

**Affiliations:** 1 MRC Integrative Epidemiology Unit, Bristol Medical School (Population Health Sciences), University of Bristol, Oakfield House, Oakfield Grove, Bristol BS8 2BN, UK

**Keywords:** cardiovascular disease, DNA methylation, causal inference, Mendelian randomization, epigenome-wide association studies

## Abstract

The extent to which genetic influences on cardiovascular disease risk are mediated by changes in DNA methylation levels has not been systematically explored. We developed an analytical framework that integrates genetic fine mapping and Mendelian randomization with epigenome-wide association studies to evaluate the causal relationships between methylation levels and 14 cardiovascular disease traits. We identified ten genetic loci known to influence proximal DNA methylation which were also associated with cardiovascular traits after multiple-testing correction. Bivariate fine mapping provided evidence that the individual variants responsible for the observed effects on cardiovascular traits at the *ADCY3* and *ADIPOQ* loci were potentially mediated through changes in DNA methylation, although we highlight that we are unable to reliably separate causality from horizontal pleiotropy. Estimates of causal effects were replicated with results from large-scale consortia. Genetic variants and CpG sites identified in this study were enriched for histone mark peaks in relevant tissue types and gene promoter regions. Integrating our results with expression quantitative trait loci data, we provide evidence that variation at these regulatory regions is likely to also influence gene expression levels at these loci.

## Introduction

Approximately 88% of trait-associated variants detected by genome-wide association studies (GWASs) reside in non-coding regions of the genome and might act through gene regulation.^1^ Recent studies have incorporated data on genetic variants associated with gene expression (expression quantitative trait loci [eQTLs]) into results from GWASs of complex traits to help identify the putative causal variant in a genomic region, as well as provide evidence suggesting which genes might be influenced by this variant.^2^, ^3^, ^4^, ^5^ This direction of inquiry can be extended to other “-omic” data types to gain further insights into the mechanistic pathway between genetic variant and causally associated trait. In this study, we introduce an alternative analytical framework to integrate genetic predictors of DNA methylation levels with complex traits to evaluate bi-directional causal relationships.

DNA methylation is an epigenetic regulation mechanism that has been shown to play a key role in many biological processes and disease susceptibility.^6^, ^7^, ^8^ Recent studies have had success in identifying genetic variants associated with DNA methylation (methylation quantitative trait loci [mQTLs]) and report that they appear to overlap with eQTLs at a large number of loci across the genome.^9^, ^10^ This suggests that both DNA methylation and gene expression could reside along the causal pathway between genetic variation and disease, although thus far, uncovering evidence of a mediated effect between mQTLs and traits has been more limited than using eQTLs.^11^, ^12^, ^13^, ^14^ Identifying epigenetic markers for disease risk should prove valuable in understanding the underlying biological mechanisms for trait-associated variants.^15^ Indeed, the value of this approach was demonstrated in a recent study that applied the SMR^2^ method to uncover pleiotropic effects between methylation levels and a range of complex traits.^16^

Mendelian randomization (MR) is a method by which genetic variants robustly associated with modifiable exposures can be used as instrumental variables to infer causality among correlated traits.^17^, ^18^ If DNA methylation resides along the causal pathway between genetic variant and trait, we would expect it to be correlated with our trait of interest. However, much like other traits analyzed in epidemiological studies, DNA methylation is prone to confounding and reverse causation. Using an MR framework, we can investigate whether DNA methylation has a causal relationship with a phenotypic outcome, suggesting that it might reside along the causal pathway to disease.^19^ Effects such as this can be referred to as “mediation,” as DNA methylation is mediating the effect from genetic variant to phenotype along the same biological pathway. As discussed in a recent review, MR has advantages over alternative approaches in mediation analysis (such as the causal inference test^20^), as it can detect the correct direction of effect in the presence of measurement error.^21^ It is important to note that all current methods are faced with the challenge of distinguishing mediation from horizontal pleiotropy, defined as effects where genetic variation influences multiple phenotypes simultaneously^22^ (such as DNA methylation and a complex trait) via independent biological pathways.

Recent approaches to MR have shown that the robustness of causal inference is improved if there are many instruments because one can evaluate whether the SNP effects on the causal trait are proportional to the SNP effects on the consequential trait.^17^, ^23^ We exploit this property to evaluate the causal influence of complex traits (which typically have many instruments) on DNA methylation (i.e., bi-directional MR^24^). A pitfall of evaluating the causal influence of DNA methylation on complex traits, however, is that DNA methylation is typically instrumented by only a single *cis*-acting variant. Hence, an unreliable MR estimate of causality could arise simply because the mQTL is in linkage disequilibrium (LD) with a variant that influences the cardiovascular trait through means other than the methylation level.

**Figure 1:**
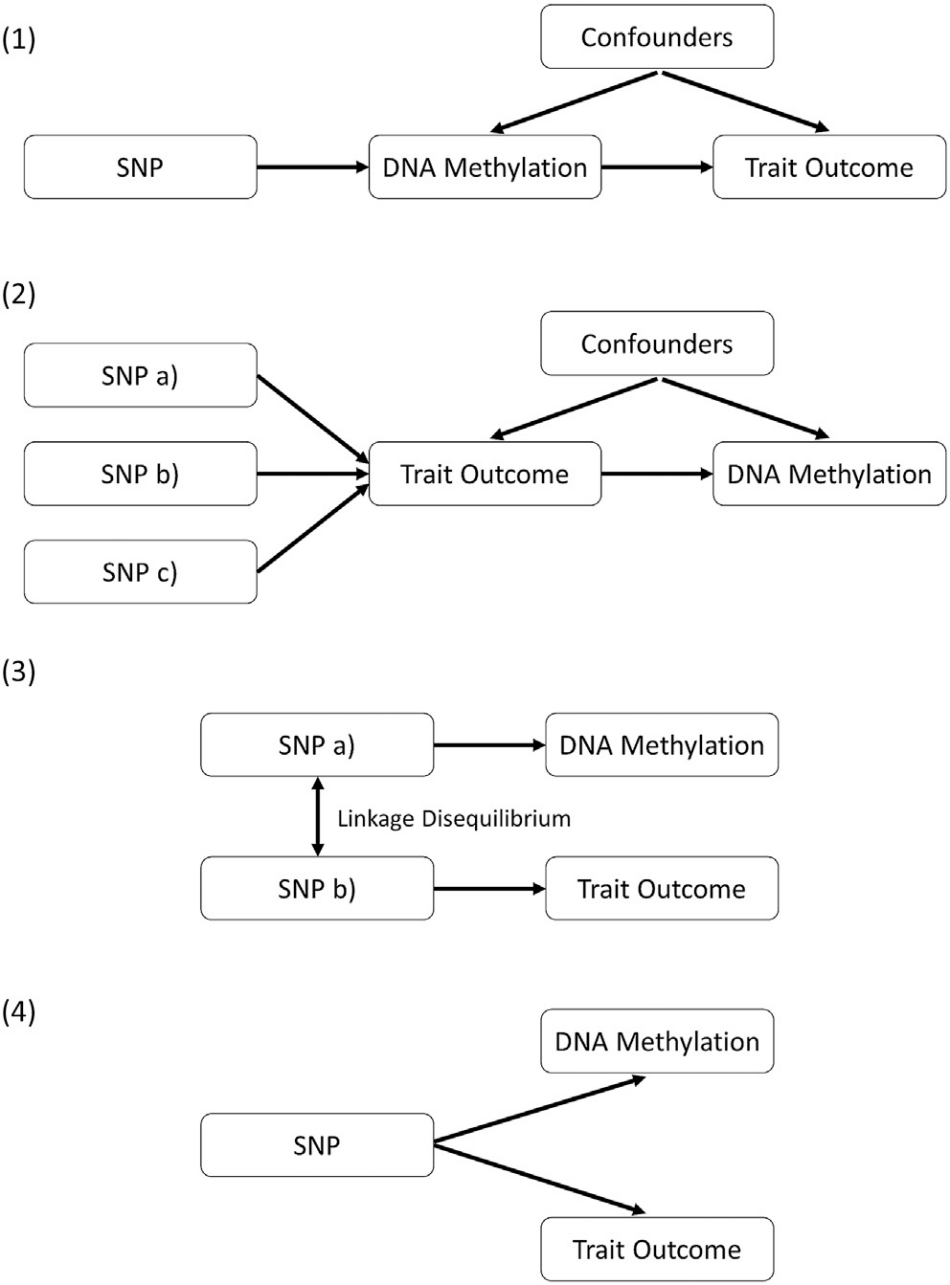
Explanations Evaluated to Explain Observed Associations between mQTLs and Trait Outcomes (1) The genetic variant has an effect on the phenotype, mediated through DNA methylation. (2) The genetic variant has an effect on the phenotype by alternative biological mechanisms, which then has a downstream effect on DNA methylation at this locus. (3) The genetic variant that influences DNA methylation is simply in LD with another variant that is influencing the associated trait. (4) The genetic variant influences both DNA methylation and phenotype by two independent biological pathways (also known as horizontal pleiotropy).

Together, the causal relationships between DNA methylation and cardiovascular traits are delineated into four potential categories (Figure 1).

1. The genetic variant has an effect on the phenotype, mediated by DNA methylation.
2. The genetic variant has an effect on the phenotype by alternative biological mechanisms, which then has a downstream effect on DNA methylation at this locus.
3. The genetic variant that influences DNA methylation is simply in LD with another variant that is influencing the associated trait.
4. The genetic variant influences both DNA methylation and phenotype by two independent biological pathways (also known as horizontal pleiotropy).

To address this issue, in this study we developed and implemented a framework that integrates MR with fine mapping to evaluate the likelihood that the mQTL is the same causal variant as the SNP influencing the cardiovascular trait. Other colocalization methods using intermediate traits have been devised for this purpose,^2^, ^25^, ^26^ including the joint likelihood mapping (JLIM) method,^27^ which we used to support our findings. We also undertook functional informatics and incorporated eQTL data because these can support findings suggesting that DNA methylation resides on the causal pathway between variant and disease. However, a limitation of using single-variant instruments in general is that it is not possible to reliably distinguish horizontal pleiotropy from mediation.^28^

In our discovery analysis, we used genotype and DNA methylation data from prepubertal individuals to discover causal pathways on early childhood phenotypes. Replication was then undertaken with GWAS summary statistics from large-scale consortia.

## Material and Methods

### The Avon Longitudinal Study of Parents and Children (ALSPAC)

ALSPAC is a population-based cohort study investigating genetic and environmental factors that affect the health and development of children. The study methods are described in detail elsewhere.^29^, ^30^ In brief, 14,541 pregnant women residents in the former region of Avon, UK, with an expected delivery date between April 1, 1991 and December 31, 1992, were eligible to take part in ALSPAC. Detailed information and biosamples have been collected on these women and their offspring at regular intervals, which are available through a searchable data dictionary.

Written informed consent was obtained for all study participants. Ethical approval for the study was obtained from the ALSPAC Ethics and Law Committee and the Local Research Ethics Committees.

### Accessible Resource for Integrative Epigenomic Studies Project (ARIES)

#### Samples

Blood samples were obtained for 1,018 ALSPAC mother-offspring pairs (mothers at two time points and their offspring at three time points) as part of the Accessible Resource for Integrative Epigenomic Studies project (ARIES).^31^ The Illumina HumanMethylation450 (450K) BeadChip array was used to measure DNA methylation at over 480,000 sites across the epigenome.

#### Methylation Assays

DNA samples were treated with bisulfite with the Zymo EZ DNA Methylation Kit (Zymo). The Illumina HumanMethylation450 BeadChip (HM450k) was used to measure methylation across the genome, and the following arrays were scanned by Illumina iScan, as well as reviewed for quality by GenomeStudio. A purpose-built laboratory information management system (LIMS) was responsible for generating batch variables during data generation. LIMS also reported quality control (QC) metrics for the standard probes on the HM450k for all samples and excluded those that failed QC. We also excluded data points with a read count of 0 or with a low signal-to-noise ratio (p value > 0.01) on the basis of the QC report from Illumina to maintain the integrity of probe measurements. We then compared methylation measurements across time points for the same individual and with SNP-chip data (HM450k probes clustered by k-means) to identify and remove sample mismatches. All remaining data from probes were normalized with the Touleimat and Tost^32^ algorithms in R with the wateRmelon package.^33^ Then we rank-normalized the data to remove outliers. We removed potential batch effects by regressing data points on all covariates. These included the bisulfite-converted DNA (BCD) plate batch and white blood cell count, which was adjusted for with the “estimateCellCounts” function in the minfi Bioconductor package.^34^

#### Genotyping Assays

Genotype data were available for all ALSPAC individuals enrolled in the ARIES project, which had previously undergone quality control, cleaning, and imputation at the cohort level. ALSPAC offspring selected for this project had previously been genotyped with the Illumina HumanHap550 quad genome-wide SNP genotyping platform (Illumina) by the Wellcome Trust Sanger Institute (WTSI, Cambridge, UK) and the Laboratory Corporation of America (LCA). Samples were excluded on the basis of incorrect sex assignment, abnormal heterozygosity (<0.320 or >0.345 for WTSI data; <0.310 or >0.330 for LCA data), high missingness (>3%), cryptic relatedness (>10% identity by descent), and non-European ancestry (detected by multidimensional scaling analysis). After QC, 500,527 SNP loci were available for the directly genotype dataset.

#### Imputation

Imputation was performed with a joint reference panel of variants discovered through whole-genome sequencing (WGS) in the UK10K project^35^ along with known variants taken from the 1000 Genomes reference panel. We developed additional functionality in IMPUTE2^36^ so we could use each reference panel to impute missing variants in their counterparts before ultimately combining them together. Following Gaunt et al.,^8^ before imputation we performed strict filtering by using Hardy-Weinberg equilibrium p > 5 × 10^−7^ and minor allele frequency (MAF) > 0.01. After imputation, we converted the dosages to best-guess genotypes and filtered to keep only variants with an imputation quality score ≥ 0.8 and MAF > 0.01.

#### Phenotypes

The 14 phenotypes analyzed in this study are as follows. At the ALSPAC clinic, subjects aged 7 years (mean age: 7.5, range: 7.1–8.8) were measured; height was measured to the nearest 0.1 cm with a Harpenden stadiometer (Holtain Crosswell), and weight was measured to the nearest 0.1 kg on Tanita electronic scales. Body mass index (BMI) was calculated as (weight [kg])/(height [m]).^2^ Blood pressure was measured with a Dinamap 9301 vital monitor using the appropriate cuff size by trained staff. Two readings of both systolic and diastolic blood pressure (SBP and DBP, respectively) were taken when the study participants were at rest, and the mean of each was used as a measurement in our analysis.

Non-fasting blood samples were taken from participants who attended the clinic at age 10 years (mean age: 9.9, range: 8.9–11.5). Plasma lipid concentrations (total cholesterol [TC], triglycerides [TG], and high-density lipoprotein cholesterol [HDL]) were measured by modification of the standard Lipid Research Clinics Protocol with enzymatic reagents for lipid determination.^37^ Low-density lipoprotein cholesterol (LDL) concentration was subsequently calculated with the Friedwald equation^38^ as follows:

LDLc=TC−(HDLc+TG×0.45)

Very-low-density lipoprotein cholesterol (VLDL) concentration was calculated as follows:

VLDLc=TC−(HDLc+LDLc)

Apolipoprotein A (Apo A1) and apolipoprotein b (Apo B) were measured by immunoturbidimetric assays (Roche). Interleukin 6 (IL-6) and adiponectin were measured by enzyme-linked immunosorbent assay (R&D Systems). High-sensitivity C-reactive protein (CRP) was measured by an automated particle-enhanced immunoturbidimetric assay (Roche). Leptin was measured in house by a linked immunosorbent assay that had been validated against commercial methods.^39^ All assay coefficients of variation were <5%.

### Statistical Analysis

We undertook an mQTL-wide association study (MWAS) to evaluate the association between variants known to influence DNA methylation (referred to hereafter as mQTL) and each trait in turn. This was decided over a conventional epigenome-wide association study (EWAS) (i.e., evaluating the association between methylation levels at CpG sites and traits) given that ALSPAC had a larger proportion of individuals with genotype data than with 450K data after phenotypes were merged.

All mQTLs previously identified in ARIES were considered for this analysis, and the methods have been described in detail previously.^8^ In brief, to discover mQTLs, Gaunt et al.^8^ used a linear regression model adjusted for age, sex, bisulphite conversion batch, the top ten ancestry principal components, and cell counts to evaluate the associations of 8,074,398 imputed genetic variants against each of the 395,625 eligible methylation probes. We filtered methylation probes for exclusion on the basis of evaluations by Naeem et al.^40^ by using their criteria of overlapping SNPs at CpG probes, probes that map to multiple locations and repeats on the 450K array. We applied a conservative multiple-testing correction to define mQTLs (p < 1.0 × 10^−14^). This threshold was selected because it equates to a false-positive rate of 0.2% after a Bonferroni correction is applied to account for the number of tests undertaken previously in ARIES. Furthermore, this strict threshold reduces the risk of MR analyses suffering from weak instrument bias. Full details on the mQTL analysis can be found in the study by Gaunt et al.^8^

The mQTL discovery study used the COJO-slct routine in GCTA to identify independent mQTLs, which was important to ensure that variants used as instruments were independent for downstream MR analyses. We excluded mQTLs associated with a CpG site that was more than 1 Mb away (known as *trans*-mQTLs), therefore leaving mQTLs that were associated only with a nearby CpG site (known as *cis*-mQTLs). This was to reduce the possibility of pleiotropy in our analysis given that variants associating with methylation at multiple CpG sites across the epigenome might influence independent biological pathways simultaneously. This left 37,812 independent mQTL eligible for analysis.

The mQTLs were analyzed sequentially with each trait by linear regression with adjustment for age and sex. We also performed a sensitivity analysis adjusting for the first ten principal components to evaluate whether population stratification was influencing our results in this analysis, although we did not anticipate this given previous evaluations of population structure in the ALSPAC cohort.^41^ Results were plotted on a Manhattan plot with code derived from the qqman R package.^42^ Scripts to generate this plot are available at the location specified in the Web Resources.

### Mendelian Randomization Analysis

Observed associations between genotype and traits that survived a stringent multiple-testing threshold (i.e., p < 0.05/number of tests undertaken) were then analyzed by MR. We performed this analysis to estimate the potential causal effect of DNA methylation on cardiovascular traits, given that we anticipated observing evidence of association after having already undertaken an MWAS. MR was undertaken by two-stage least-squares (2SLS) regression with DNA methylation as our exposure, phenotypic trait as our outcome, and the relevant mQTL as our instrumental variable. Measures of DNA methylation were initially taken from the childhood time point in ARIES (mean age, 7.5 years; standard deviation, 0.15) because this was the closest time point to phenotype measurements. Follow-up analyses were also undertaken with methylation data from the birth time point (with cord blood) and the adolescent time point (mean age, 17.1 years; standard deviation, 1.01). We used the R package “systemfit”^43^ to obtain causal effect estimates with 2SLS.

We replicated observed effects by undertaking a two-sample MR analysis (2SMR)^44^ with estimated effects between genetic variants and associated traits obtained from published studies. Moreover, a two-sample framework removes any potential bias encountered in the discovery analysis as a result of the existence of effects on both methylation and traits in the same sample. When observed effects for sentinel mQTL were not available from published studies, we used variants in LD with these SNPs instead (r^2^ > 0.8).

**Figure 2:**
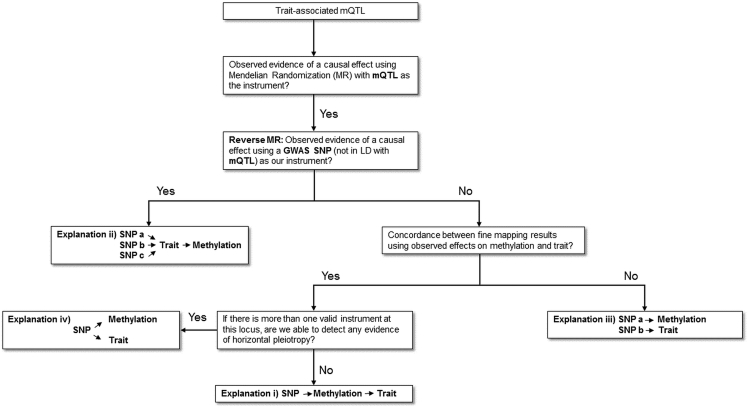
Analysis Pipeline to Evaluate Explanations for Observed Associations between mQTLs and Trait Outcomes This flowchart provides an overview of the analysis plan in this study for evaluating four different explanations that might explain trait-associated mQTLs.

Figure 1 illustrates the four possible explanations investigated where evidence of a causal effect was observed by MR. Figure 2 provides an overview of our approach to investigate these explanations. To robustly test explanation 2, we performed the reverse MR analysis, evaluating whether the cardiovascular trait influenced DNA methylation levels at the CpG site of interest. Instruments for this analysis were identified with the NHGRI-EBI GWAS Catalog.^45^ Relevant GWASs for IL-6 were not available at the time of analysis and so we identified instruments on the basis of the findings from Naitza et al.^46^ (p < 5.0 × 10^−8^). A p value greater than 0.05 indicated that explanation 2 was unlikely in each instance.

### Bivariate Fine Mapping

Bivariate fine mapping was undertaken with FINEMAP^47^ at each locus detected in the previous analysis. For each variant at a locus, FINEMAP generates a Bayes factor that reflects the likelihood that the variant is the underlying causal variant at this region. Bivariate fine mapping requires all variants at a locus to be fine mapped with two different effect estimates: (1) observed effects between SNPs and DNA methylation and (2) observed effects between SNPs and outcome phenotypes. Given that we initially pruned all mQTL effects to identify independent loci, we included only variants that were in high LD (r^2^ ≥ 0.8) with the sentinel SNP for each association signal before applying FINEMAP with default settings. Interpretation of these results is therefore based on at least one underlying causal variant at each loci, given that follow-up analyses are necessary for evaluating whether multiple causal variants might be contributing to observed effects. Posterior probabilities to reflect the likelihood of multiple causal variants were calculated with FINEMAP.

We performed this analysis to evaluate explanation 3, that the mQTL analyzed might simply be in LD with the putative causal variant for the phenotypic trait. This was necessary because when the relationship between DNA methylation at a CpG site and the outcome trait is evaluated, there could be only one valid instrumental variable (i.e., the mQTL at this region). Bivariate fine mapping in this instance therefore evaluates whether the causal mQTL at a locus is likely to be the same causal variant for the observed effect on the outcome trait. However, it does not rule out the possibility that a single variant influences DNA methylation and an outcome trait through independent biological pathways (i.e., explanation 4).

Concordance between the top SNPs for the two sets of fine-mapping analyses would suggest that explanation 1 might be responsible for the observed effect and that DNA methylation resides on the causal pathway between variant and phenotypic trait. Bivariate fine mapping using effect estimates for both methylation and cardiovascular traits was advantageous in this study because we were able to obtain estimates for all SNPs in our dataset without having to rely on summary statistics. The concordance rate was defined as identifying the same variant from both analyses as causal after accounting for chance. We achieved this by identifying the rank of the top variant from the methylation-based analysis in the list of variants from the cardiovascular-trait analysis and then dividing that rank by the total number of variants in the region. A concordance rate < 0.05 suggested that explanation 3 was unlikely. To further evaluate explanation 3, we also used the JLIM approach.^27^ Although JLIM doesn’t specify the likely causal variant at a region, it can be used to examine whether the underlying causal variation is responsible for the observed effects on both methylation and cardiovascular traits in a two-sample framework. Prior probabilities were not integrated into these analyses with FINEMAP, which allowed for a more direct comparison with results of the JLIM method.

### Impact of mQTLs on Gene Expression and Histone Modification

We applied 2SMR to evaluate the relationship between methylation and expression by using observed effects between SNPs and expression in relevant tissue types from the Genotype-Tissue Expression (GTEx) Consortium.^48^ When observed effects for sentinel mQTLs were not available from GTEx, we identified a surrogate SNP instead (r^2^ > 0.8).

We also assessed whether any mQTLs were in LD (r^2^ > 0.8) with any previously reported histone quantitative trait loci (hQTLs).^49^ When this was true, we applied 2SMR to evaluate the causal relationship between methylation and histone modification at these loci. This analysis was for exploratory purposes because some aspects of the relationship between DNA methylation and histone modification remain unexplored, despite progress by recent studies.^50^, ^51^

### Functional Informatics

We applied the Variant Effect Predictor (VEP)^52^ to the top-ranked mQTLs from the bivariate fine-mapping analysis to calculate their predicted consequence. We obtained enhancer annotations from the Illumina 450K annotation file and additional regulatory data from Ensembl^53^ to evaluate whether mQTLs and CpG sites were located within regulatory regions of the genome. Because we were interested in cardiovascular and lipid traits in this study, we used tissue-specific data from the Roadmap Epigenomics Project^54^ to infer whether the potential causal variants and CpG sites at each locus resided within histone mark peaks and regions of DNase hypersensitivity. These tissues were adipose-derived mesenchymal stem cells, adipose nuclei, aorta, fetal heart, left ventricle, right atrium, and right ventricle, which we selected because of their biological relevance in cardiovascular etiology.

We performed enrichment analysis to test whether lead SNPs and associated CpG sites were located in regulatory regions more than can be accounted for by chance. To calibrate background expectations, we obtained matched SNPs by using snpSNAP^55^ and identified matched CpG sites by randomly sampling 450K array probes that were in similar regions across the genome (i.e., within CpG islands or first exons, etc.). We investigated enrichment by using the hypergeometric test and accounted for multiple testing for by randomly sampling control SNPs and probes and re-running analyses for 10,000 iterations.

## Results

### Mining for Putative Causal Influences of Methylation on Cardiovascular Traits

**Figure 3:**
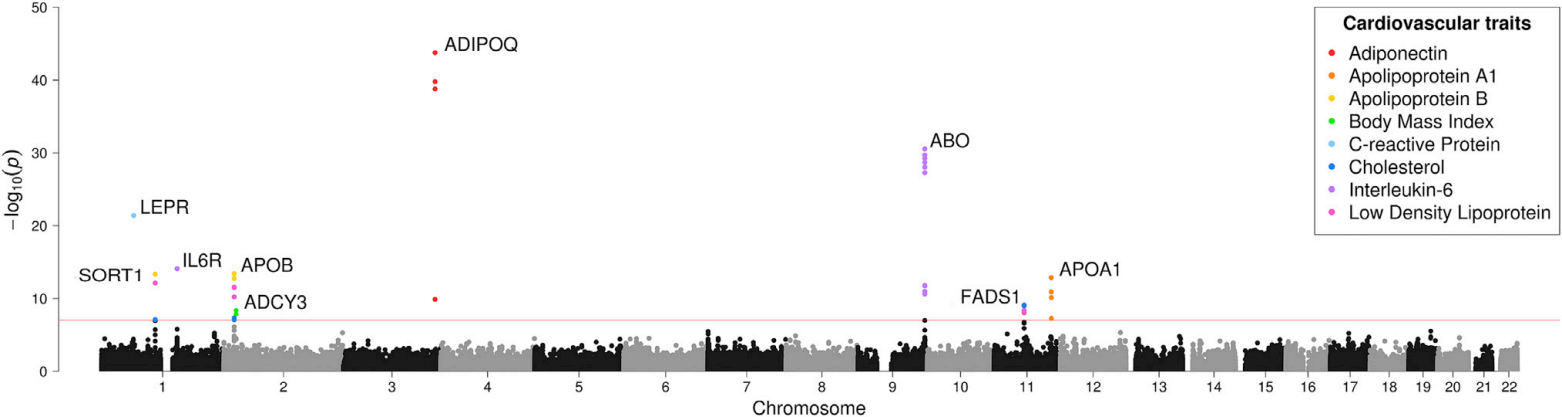
Manhattan Plot Illustrating Observed Association between mQTLs and Cardiovascular Traits Points represent –log10 p values (y axis) for genetic variants according to their genomic location (x axis). Effects that survived the multiple-testing threshold in our analysis (p < 9.45 × 10^−8^ – represented by the red horizontal line) are colored according to their associated trait and annotated according to the most likely affected gene.

We undertook 529,368 tests to evaluate the association between previously identified mQTLs in ARIES with each trait in turn (37,812 unique variants × 14 traits). We identified ten independent association signals, which, after multiple-testing correction, provided strong evidence of association (p < 9.45 × 10^−8^ [i.e., 0.05/529,368]); these can be found in Table 1 and Figure 3. Two of these effects were observed at the same CpG site near *ADIPOQ* (MIM: 612556), although they were identified with two independent mQTLs (r^2^ = 0.02).

**Table 1:** Results of Linear Regression Analysis between Genetic Variants and Traits

**SNP**	**Gene**	**CpG**	**Trait**	**Sample Size**	**Beta**	**SE**	**p Value**	**% Explained**
rs266772	*ADIPOQ*	cg05578595	adiponectin (ng/mL)	4,248	−0.992	0.070	1.72 × 10^−44^	4.51%
rs687621	*ABO*	cg21160290	IL-6 (pg/mL)	4,241	−0.265	0.022	1.15 × 10^−31^	3.05%
rs13375019	*LEPR*	cg04111102	CRP (mg/L)	4,251	−0.213	0.022	2.65 × 10^−22^	2.20%
rs7549250	*IL6R*	cg02856953	IL-6 (pg/mL)	4,241	−0.176	0.022	9.71 × 10^−16^	1.40%
rs169109	*ADIPOQ*	cg05578595	adiponectin (ng/mL)	4,248	−0.167	0.022	1.44 × 10^−14^	1.34%
rs541041	*APOB*	cg25035485	Apo B (g/L)	4,251	−0.209	0.028	3.76 × 10^−14^	1.32%
rs7528419	*SORT1*	cg00908766	Apo B (g/L)	4,251	−0.196	0.026	4.63 × 10^−14^	1.30%
rs625145	*APOA1*	cg04087571	Apo A1 (g/L)	4,251	0.200	0.027	9.78 × 10^−14^	0.94%
rs174544	*FADS1*	cg19610905	total cholesterol (mmol/L)	4,250	−0.143	0.023	8.61 × 10^−10^	0.86%
rs6749422	*ADCY3*	cg01884057	BMI	6,076	0.109	0.018	1.28 × 10^−9^	0.55%

Abbreviations are as follows: SNP, single-nucleotide polymorphism; gene, most likely affected gene; CpG, 450K probe ID; trait, associated trait; sample size, sample size for this effect; beta, observed effect size (units in standard deviations); SE, standard error of the effect size; p value, p value for observed effect; and % explained, proportion of trait variance explained by mQTLs.

The ten sentinel mQTLs identified in this analysis were strongly associated with DNA methylation only at a proximal CpG site and not any other CpG sites in the epigenome, according to our findings in ARIES. A summary of these mQTLs can be found in Table S1. We repeated our analysis with adjustment for the first ten principal components, although results did not suggest that population stratification was an issue in this analysis (Table S2).

### Inferring Putative Causal Relationships

We obtained estimates of putative causal effects between methylation and cardiovascular traits at each locus in the MR analysis by using mQTLs as our instrumental variables (Table 2). Effect estimates suggested a direct relationship between methylation and cardiovascular traits at the *IL6R* (MIM: 147880), *APOB* (MIM: 107730), *SORT1* (MIM: 602458), and *ADCY3* (MIM: 600291) loci (i.e., increased methylation results in an observed increase in the cardiovascular trait), whereas an inverse relationship was observed at the *ADIPOQ*, *ABO* (MIM: 110300), *LEPR* (MIM: 601007), *APOA1* (MIM: 107680), and *FADS1* (MIM: 606148) loci (i.e., increased methylation causes a decrease in cardiovascular-trait levels). Because two independent mQTLs were contributing to methylation at *ADIPOQ*, we undertook multivariate MR, which provided strong evidence of an inverse relationship between methylation and adiponectin at this locus (−0.548 ng/mL per standard deviation change in methylation levels, standard error = 0.107, p = 3.79 × 10^−7^).

**Table 2:** Results of MR Analysis between DNA Methylation and Traits

**SNP**	**Gene**	**CpG**	**Trait**	**Sample Size**	**Beta**	**SE**	**p Value**
rs266772	*ADIPOQ*	cg05578595	adiponectin (ng/mL)	646	−0.846	0.168	5.93 × 10^−7^
rs687621	*ABO*	cg21160290	IL-6 (pg/mL)	646	−0.293	0.061	1.77 × 10^−6^
rs13375019	*LEPR*	cg04111102	CRP (mg/L)	646	−0.265	0.076	0.001
rs7549250	*IL6R*	cg02856953	IL-6 (pg/mL)	646	0.468	0.175	0.008
rs169109	*ADIPOQ*	cg05578595	adiponectin (ng/mL)	646	−0.363	0.121	0.003
rs541041	*APOB*	cg25035485	Apo B (g/L)	646	0.298	0.114	0.009
rs7528419	*SORT1*	cg00908766	Apo B (g/L)	646	0.271	0.064	2.74 × 10^−5^
rs625145	*APOA1*	cg04087571	Apo A1 (g/L)	646	−0.301	0.082	2.68 × 10^−4^
rs174544	*FADS1*	cg19610905	total cholesterol (mmol/L)	646	−0.363	0.121	0.003
rs6749422	*ADCY3*	cg01884057	BMI	846	0.106	0.048	0.028

Abbreviations are as follows: SNP, single-nucleotide polymorphism; gene, most likely affected gene; CpG, 450K probe ID; trait, associated trait; sample size, sample size for this effect; beta, observed effect size (units in standard deviations); SE, standard error of the effect size; and p value, p value for observed effect.

Taking these putative associations forward, we evaluated the potential for reverse causal relationships by performing MR of the cardiovascular traits against the DNA methylation levels by using SNPs from GWASs as our instruments. There was no evidence to suggest that the putative associations were due to the cardiovascular traits influencing the methylation levels (Table S3), and therefore these effects cannot be attributed to explanation 2. We note, however, that statistical power to detect an effect in this direction is low.

Using methylation data from two other time points across the life course (at birth and adolescence [mean age: 17.1 years]), we observed directions of effect consistent with those observed with data from the childhood time point (mean age: 7.5 years) (Tables S4 and S5). We observed evidence of association at each locus in this analysis except when we used cord data for the *ABO* and *IL6R* loci. We reproduced similar effects for nine of the ten mQTLs on cardiovascular traits by using effect estimates from published studies (Table 3). The only locus for which we were not able to find a replication effect estimate was the mQTL at *IL6R*, because it was not in LD (r^2^ > 0.8) with any previously published findings for IL-6.

**Table 3:** Results of Replication Analysis via Two-Sample MR

**SNP**	**Gene**	**Trait**	**CpG**	**CpG Effect (SE)**	**Trait Effect (SE)**	**2SMR Effect (SE)**	**p Value**	**Study**
rs266772	*ADIPOQ*	Adiponectin (ng/mL)	cg05578595	0.982 (0.103)	−0.629 (0.143)	−0.641 (0.160)	6.50 × 10^−5^	UK10K Consortium (TwinsUK individuals only)^35^
rs687621	*ABO*	IL-6 (pg/mL)	cg21160290	0.912 (0.036)	−0.245 (0.026)	−0.269 (0.03)	9.16 × 10^−19^	Naitza et al.^46^
rs2211651^∗^	*LEPR*	CRP (mg/L)	cg04111102	0.682 (0.036)	−0.170 (0.022)	−0.249 (0.035)	3.09 × 10^−13^	Reiner et al.^56^
rs541041	*APOB*	Apo B (g/L)	cg25035485	0.627 (0.053)	0.098 (0.013)	0.156 (0.025)	2.05 × 10^−10^	Kettunen et al.^57^
rs169109	*ADIPOQ*	Adiponectin (ng/mL)	cg05578595	0.383 (0.036)	−0.052 (0.005)	−0.136 (0.017)	2.58 × 10^−15^	Dastani et al.^58^
rs7528419	*SORT1*	Apo B (g/L)	cg00908766	−0.980 (0.037)	−0.089 (0.012)	0.091 (0.013)	9.20 × 10^−13^	Kettunen et al.^57^
rs625145	*APOA1*	Apo A1 (g/L)	cg04087571	−0.884 (0.044)	0.057 (0.013)	−0.064 (0.015)	1.84 × 10^−5^	Kettunen et al.^57^
rs174544	*FADS1*	total cholesterol (mmol/L)	cg19610905	−0.655 (0.031)	0.047 (0.004)	−0.072 (0.007)	9.73 × 10^−25^	Willer et al.^59^
rs6749422	*ADCY3*	BMI	cg01884057	0.908 (0.026)	0.068 (0.007)	0.075 (0.008)	8.05 × 10^−21^	Felix et al.^60^

Abbreviations are as follows: SNP, single-nucleotide polymorphism; gene, most likely affected gene; trait, associated trait; CpG, 450K probe ID; CpG effect, effect estimate of SNP on methylation; trait effect, effect estimate of SNP on trait; 2SMR effect, effect estimates from two-sample MR analysis; p value, p value for observed effect; study, published study where effect estimates for traits were obtained; and SE, standard error. The asterisk indicates that a surrogate variant was used (r^2^ > 0.8).

### Evaluating Putative Causal Variants to Infer Mediated Effects

There was concordance among the top SNPs in the bivariate fine-mapping analyses for IL-6 (*ABO* locus), BMI (*ADCY3* locus), and adiponectin (*ADIPOQ* common locus), given that the variant with the largest Bayes factor was the same for the effect on DNA methylation and outcome trait (Tables S6). These results lend support to the hypothesis that DNA methylation resides on the causal pathway between genetic variants and outcome traits (i.e., explanation 1). There was a lack of concordance for the results for adiponectin (*ADIPOQ* low-frequency locus), Apo B (*SORT1* locus), total cholesterol (*FADS1* locus), and CRP (*LEPR* locus), suggesting that the mQTLs might be in LD with the putative causal variant for the phenotypic trait (i.e., explanation 3). Results of the JLIM method supported evidence at the *ADIPOQ* and *ADCY3* loci, although we were unable to further evaluate signals at the *ABO* and *IL6R* regions because GWAS summary results were unavailable for IL-6 (Table S7). Posterior probabilities from FINEMAP suggested that there was most likely only a single variant influencing trait variation for each observed effect (Table S7).

### Investigating the Role of DNA Methylation in Gene Expression and Histone Modification

To further dissect the relationship between DNA methylation and complex traits, we sought to evaluate the influence of the methylation levels on local gene expression. Using data from the GTEx Consortium, we observed evidence of a causal relationship between methylation and expression at eight of the ten loci (Table 4). Effect estimates suggest an inverse relationship (i.e., increased methylation results in decreased gene expression) at the *ADIPOQ* (low-frequency signal) and *APOA1* loci, whereas a direct relationship was observed at the other six loci (i.e., increased methylation results in increased gene expression). We were unable to identify a surrogate variant (r^2^ > 0.8) to obtain a suitable effect estimate at the *LEPR* and *ADIPOQ* (common signal) loci.

**Table 4:** Results of Analysis Investigating Causal Relationship between Methylation and Expression via Two-Sample MR

**SNP**	**Gene**	**CpG**	**CpG Effect (SE)**	**eQTL Effect (SE)**	**eQTL p Value**	**eQTL Tissue**	**2SMR (SE)**	**p Value**
rs116552240^∗^	*ABO*	cg21160290	0.912 (0.036)	0.548 (0.069)	1.316 × 10^−13^	adipose	0.601 (0.079)	3.28 × 10^−14^
rs6737082	*ADCY3*	cg01884057	0.908 (0.026)	0.208 (0.047)	1.456 × 10^−5^	adipose	0.229 (0.052)	1.13 × 10^−5^
rs266772	*ADIPOQ*	cg05578595	0.982 (0.103)	−0.339 (0.078)	1.893 × 10^−5^	adipose	−0.345 (0.087)	7.67 × 10^−5^
rs688456	*APOA1*	cg04087571	−0.884 (0.044)	0.420 (0.095)	1.789 × 10^−5^	heart	−0.475 (0.11)	1.58 × 10^−5^
rs541041	*APOB*	cg25035485	−0.627 (0.053)	−0.370 (0.066)	6.326 × 10^−8^	heart	0.590 (0.116)	4.06 × 10^−7^
rs646776	*SORT1*	cg00908766	−0.980 (0.037)	−1.240 (0.105)	1.556 × 10^−20^	liver	1.265 (0.117)	4.01 × 10^−27^
rs174559	*FADS1*	cg19610905	−0.655 (0.031)	−0.707 (0.089)	5.629 × 10^−13^	pancreas	1.079 (0.145)	1.04 × 10^−13^
rs10908837	*IL6R*	cg02856953	−0.303 (0.039)	−0.120 (0.020)	4.171 × 10^−9^	whole blood	0.396 (0.083)	2.05 × 10^−6^

Abbreviations are as follows: SNP, single-nucleotide polymorphism; gene, most likely affected gene; CpG, 450K probe ID; CpG effect, effect estimate of SNP on methylation; eQTL effect, effect estimate of SNP on expression according to GTEx data; eQTL p, p value for eQTL from GTEx; eQTL tissue, tissue type for observed effect according to GTEx; 2SMR effect, effect estimates from two-sample MR analysis (standard deviation units per standard deviation units); p value, p value for 2SMR effect; and SE, standard error. The asterisk indicates that a surrogate variant was used (r^2^ > 0.8).

mQTLs at the *APOA1* and *IL6R* loci were also in high LD with previously reported hQTLs according to findings by Grubert et al.^49^ Results from our 2SMR analyses to evaluate the influence of methylation levels on histone modification provided strong evidence of a causal effect as well as an inverse relationship in each instance (Table S8).

### Functional Informatics

To better understand the functional role underlying these putative causal associations, we evaluated variants and CpG sites to discern whether they reside within regulatory regions across the genome. An overview of the regulatory data used can be found in Table S9. In this analysis, we used the lead variants based on the bivariate fine-mapping analysis (using effect estimates on DNA methylation) and used the VEP to predict their functional consequences (Table S10).

Every associated CpG site identified in this study resides within multiple histone mark peaks according to tissue data from the Roadmap Epigenomics Project (Table S11). All sites, with the exception of the CpG site near *ADIPOQ*, also reside in either enhancer, promoter, or promoter flanking regions. There was strong evidence of enrichment of regulatory annotations for both SNPs and CpG sites, which supports previous evidence that they are likely to have a causal downstream effect on phenotypic variation (Table S12).

## Discussion

We have designed a framework for evaluating the putative causal influences of DNA methylation on complex traits and disease via MR. For observed effects on cardiovascular traits that appear to be caused by methylation, we used bivariate fine mapping and JLIM to evaluate whether the putative causal variant influencing methylation was the same causal variant responsible for influencing the trait. The bivariate fine mapping suggested that cardiovascular traits might be influenced by altered DNA methylation levels at the *ABO*, *ADCY3*, *ADIPOQ*, *APOA1*, *APOB*, and *IL6R* regions. However, JLIM supported findings only at the *ADCY3* and *ADIPOQ* loci. This provides compelling evidence that DNA methylation might play a mediatory role for the effects at these loci. 2SMR analyses provided evidence that DNA methylation levels influenced gene expression at these loci, suggesting that functional effects for the causal variants induce a coordinated system of effects. This was important to demonstrate, given that having only single valid instruments available for CpGs meant that we were unable to robustly show that variants were not influencing methylation and traits through horizontal pleiotropy. This limitation has also been encountered by other attempts to evaluate the relationship between DNA methylation and complex traits.^16^ Nevertheless, the ability to indicate putative mediating molecular phenotypes between genetic factors and complex traits is particularly attractive for therapeutic evaluation of drug targets.

The *ABO* locus identified in this study has been associated with many different traits and diseases by previous studies,^25^, ^61^, ^62^ and there is also evidence implicating eQTLs as putative causal SNPs for this effect.^63^ Here, we provide evidence that DNA methylation might reside along the causal pathway to these observed effects (MR effect estimate: 0.29 [standard error = 0.06] change in trait per standard deviation change in methylation), although its widespread effect also raises the possibility of horizontal pleiotropy. A deletion (rs200533593) was found to be the putative causal variant for both the observed effect on DNA methylation and phenotypic variation.

The observed effect of genetic variation at *ADCY3* on BMI is a relatively recent finding.^60^, ^64^, ^65^ In this study, our bivariate fine-mapping analysis suggests that an intergenic variant (rs6737082) might be responsible for the observed signal that is mediated through DNA methylation at this locus (MR effect estimate: 0.11 [0.05]). Furthermore, a variant in LD with rs6737082 (rs713586, r^2^ = 0.80) has been previously reported to regulate DNA methylation at this location in adipose tissue.^7^

Two independent effects associated with adiponectin were detected near *ADIPOQ* in our study. The common variant signal was located upstream of *ADIPOQ* within *RFC4* but associated with DNA methylation levels proximal to *ADIPOQ*, which can help explain this variant’s observed effect on adiponectin (MR effect estimate: −0.36 [0.12]). Concordance in the bivariate fine-mapping analysis suggested that a non-coding transcript variant (rs169109) was responsible. The lead SNP from the ADIPOGen Consortium^66^ at this locus (rs6810075) is neither an mQTL nor in high LD with rs169109 (r^2^ = 0.20), suggesting that these two association signals influence adiponectin levels by alternative biological mechanisms. The low-frequency variant signal was previously detected by the UK10K project,^35^ although bivariate fine-mapping results at this locus suggest that the causal mQTL was in LD with the trait-associated variant.

The CpG site associated with Apo A1resides between *APOA1* and *APOA1-antisense* (*APOA1-AS*), a negative transcriptional regulator of *APOA1* that has been shown to increase *APOA1* expression both *in vitro* and *in vivo*.^67^ The highest ranked mQTL according to our bivariate fine mapping using estimates with DNA methylation is in a promoter region upstream of *APOA1*, suggesting that it might be more likely to influence *APOA1* than *APOA1-AS*. GWAS association signals for lipid traits have been previously reported at this locus.^68^, ^69^ However, given the evidence in this study of a causal effect with DNA methylation (MR effect estimate: −0.30 [0.08] g/L per SD methylation level), it is possible that these are downstream effects of the observed effect on Apo A1 variation.

The signal at the *IL6R* locus influencing IL-6 has been previously associated with a range of traits related to respiratory and cardiovascular health.^70^, ^71^, ^72^ Our results suggest that genetic variation at *IL6R* influences DNA methylation at this region, which in turn could have a downstream effect on the amount of IL-6 (MR effect estimate: 0.47 [0.18] pg/mL per standard deviation methylation level). Furthermore, this association signal was not in LD with a previously reported missense variant at this locus (rs2228145, r^2^ = 0.47 in ALSPAC), which was also supported by findings from an in-depth functional study of this variant.^73^

Evidence from the GTEx Consortium suggests that mQTLs at all eight of the loci with available expression data overlap eQTL effects, which serves as a form of independent replication of the relationships discovered through DNA methylation levels. It is biologically plausible that a variant’s impact on DNA methylation levels might have a downstream effect on gene expression along the causal pathway to disease,^74^, ^75^ which could help explain these observations. Effects at four loci in particular appear to be biologically plausible in this regard, as the likely genes influenced by these variants are involved in the protein synthesis of the associated trait (i.e., *ADIPOQ* with adiponectin, *APOB* with Apo B, *APOA-I* with Apo A1, and *IL6R* with IL-6). Furthermore, each CpG site identified in this study resides within histone mark peaks in adipose tissue according to data from the Roadmap Epigenomics project. There was evidence of enrichment for these observations in comparison to background CpG sites which are located in similar genomic regions.

As with any study that applies single-instrument MR to investigate causal relationships in epidemiology, an important limitation is the inability to disentangle potential horizontal pleiotropic effects, where the same causal variant influences both exposure (i.e., DNA methylation) and outcome (i.e., cardiovascular trait) through independent pathways. To reduce the possibility of this, we selected mQTLs that were influencing only proximal CpG sites and not others in the epigenome, given that *trans*-mQTLs would be more prone to influence traits via alternative biological mechanisms. Although ARIES includes CpG sites that have two or three independent instruments (such as the CpG site at *ADIPOQ* in this study), distinguishing mediation from pleiotropy at these loci remains a challenging endeavor. Future studies that continue to uncover multiple mQTLs per CpG across the genome (as well as across various tissue types) should facilitate analyses that are able to more reliably address concerns of pleiotropy by using methods such as MR-Egger^76^ and median- and mode-based MR estimators.^77^, ^78^ These findings should also facilitate analyses that model the joint effects of multiple causal mQTLs at loci across the genome rather than evaluate mQTL effects independently of each other, as we did in this study.

Weak instrumental variables and reverse causation are other factors that can bias MR analyses. Our analysis is unlikely to have suffered from the former because each mQTL had a large effect on DNA methylation in *cis* (p < 1.0 × 10^−14^) and was robustly associated with traits that we were able to replicate by using results from studies with large population samples. We conducted analyses to evaluate whether reverse causation was an issue in our study (i.e., trait variation caused changes in DNA methylation at each locus). Although our results suggest that this was not the case, it is important to note that the statistical power to detect causal effects in this direction is low because the sample size available for the SNP effects on CpG levels was small.

In this study, we demonstrated the value of 2SMR to MR analyses using summary statistics.^44^, ^79^ This allowed us to provide evidence of replication for the observed effects in our study as well as investigate the relationship between DNA methylation and expression along the causal pathway to disease. This approach has the attractive advantage of enabling the interrogation of the potential epigenetic-complex trait interplay on a much wider scale by foregoing the requirement that “omic” data and phenotypes are measured in the same sample.

## Web Resources

ALSPAC, http://www.bristol.ac.uk/alspac/
ALSPAC data dictionary, http://www.bris.ac.uk/alspac/researchers/data-access/data-dictionary/
ARIES Explorer, http://www.ariesepigenomics.org.uk/ariesexplorer
Ensembl, http://www.ensembl.org/index.html
GTEx Portal, http://www.gtexportal.org/home/
GWAS Catalog, http://www.ebi.ac.uk/gwas/
Multicolored Manhattan plot script, https://github.com/MRCIEU/qqman_multiple_colours
mQTL Database, http://www.mqtldb.org/
OMIM, http://www.omim.org/
Roadmap Epigenomics, http://www.roadmapepigenomics.org/
SNPsnap, http://www.broadinstitute.org/mpg/snpsnap/index.html

## Supplemental Data

Document S1. Tables S1–S8

Spreadsheet S1. Tables S9–S12

Document S2. Article plus Supplemental Data
