# Supplementary material for: Mendelian Randomization Analysis Identifies CpG Sites as Putative Mediators for Genetic Influences on Cardiovascular Disease Risk
Source: Am J Hum Genet. 2017 Oct 5;101(4):590–602. doi: 10.1016/j.ajhg.2017.09.003 (PMC5630190; doi:10.1016/j.ajhg.2017.09.003)
Supplement: Document S1. Tables S1–S8 [file mmc1.pdf]

The American Journal of Human Genetics, Volume *101*

Supplemental Data

Mendelian Randomization Analysis Identifies CpG Sites as Putative Mediators for Genetic Influences on Cardiovascular Disease Risk

Tom G. Richardson, Jie Zheng, George Davey Smith, Nicholas J. Timpson, Tom R. Gaunt, Caroline L. Relton, and Gibran Hemani

SNP	Gene	Chr	BP	MAF	Other	Effect
rs266772	*ADIPOQ*	*3*	186546540	0.027	T	C
rs687621	*ABO*	*9*	136137065	0.328	G	A
rs13375019	*LEPR*	*1*	66123136	0.367	C	G
rs7549250	*IL6R*	*1*	154404336	0.416	C	T
rs169109	*ADIPOQ*	*3*	186525798	0.496	G	C
rs541041	*APOB*	*2*	21294975	0.181	G	A
rs7528419	*SORT1*	*1*	109817192	0.212	G	A
rs625145	*APOA1*	*11*	116727936	0.198	T	A
rs174544	*FADS1*	*11*	61567753	0.298	A	C
rs6749422	*ADCY3*	*2*	25150011	0.487	G	C

Table S1: Summary of methylation quantitative trait loci used as instruments in Mendelian randomization analysis SNP – Single Nucleotide Polymorphism, Chr – Chromosome, BP – Base position, MAF – minor allele frequency, Effect – effect allele coded additively for each SNP in all analyses, Other – other allele for this SNP

SNP	Gene	CpG	Trait	Sample Size	Beta	SE	P-value
rs266772	*ADIPOQ*	cg05578595	Adiponectin	4248	-0.990	0.070	5.97×10^{-44}
rs687621	*ABO*	cg21160290	Interleukin-6	4241	-0.260	0.023	9.74×10^{-30}
rs13375019	*LEPR*	cg04111102	C-reactive protein	4251	-0.220	0.022	1.14×10^{-22}
rs7549250	*IL6R*	cg02856953	Interleukin-6	4241	-0.169	0.022	1.87×10^{-14}
rs169109	*ADIPOQ*	cg05578595	Adiponectin	4248	-0.138	0.023	2.98×10^{-10}
rs541041	*APOB*	cg25035485	Apo B	4251	-0.211	0.028	4.98×10^{-14}
rs7528419	*SORT1*	cg00908766	Apo B	4251	-0.199	0.026	5.31×10^{-14}
rs625145	*APOA1*	cg04087571	Apo A1	4251	0.204	0.027	8.00×10^{-14}
rs174544	*FADS1*	cg19610905	Cholesterol	4250	-0.141	0.023	1.96×10^{-09}
rs6749422	*ADCY3*	cg01884057	Body mass index	6076	0.107	0.018	3.36×10^{-09}

Table S2: Results of linear regression analysis between genetic variants and traits adjusting for the top 10 principal components

Tables S3

SNP	CpG site	Trait	Beta	Standard Error	P-value
rs7531118_T	cg01884057	BMI	-3.350	4.758	0.482
rs7550711_T	cg01884057	BMI	2.203	2.142	0.304
rs543874_G	cg01884057	BMI	0.668	1.394	0.632
rs13021737_A	cg01884057	BMI	0.077	0.749	0.918
rs10182181_G	cg01884057	BMI	0.106	0.048	0.028
rs13078960_G	cg01884057	BMI	-0.664	1.020	0.516
rs1516725_T	cg01884057	BMI	-1.972	2.343	0.400
rs13130484_T	cg01884057	BMI	1.152	2.586	0.656
rs2112347_G	cg01884057	BMI	0.665	1.117	0.552
rs943005_T	cg01884057	BMI	-1.696	2.152	0.431
rs2183825_C	cg01884057	BMI	-10.916	235.015	0.963
rs11030104_G	cg01884057	BMI	0.353	1.745	0.840
rs3817334_T	cg01884057	BMI	0.988	1.377	0.473
rs7138803_A	cg01884057	BMI	-3.670	5.625	0.514
rs7144011_T	cg01884057	BMI	-0.089	2.330	0.969
rs13329567_T	cg01884057	BMI	-1.057	1.076	0.326
rs3888190_A	cg01884057	BMI	1.067	2.579	0.679
rs6567160_C	cg01884057	BMI	-0.486	0.781	0.534
rs11672660_T	cg01884057	BMI	5.258	21.226	0.804

Table S3a: Mendelian randomization analysis using SNPs with evidence of association with BMI from GWAS as instrumental variables to investigate reverse causation at *ADCY3*

SNP	CpG site	Trait	Beta	Standard Error	P-value
rs1108842_C	cg05578595	Adiponectin	-0.195	0.534	0.715
rs1597466_T	cg05578595	Adiponectin	3.774	9.589	0.694
rs2062632_C	cg05578595	Adiponectin	-1.319	1.352	0.330
rs6810075_C	cg05578595	Adiponectin	-1.234	0.876	0.159
rs7615090_G	cg05578595	Adiponectin	0.231	0.533	0.665
rs2980879_A	cg05578595	Adiponectin	-5.060	19.255	0.793
rs7955516_C	cg05578595	Adiponectin	1.255	2.705	0.643
rs601339_G	cg05578595	Adiponectin	0.943	1.059	0.373
rs7964945_A	cg05578595	Adiponectin	-0.600	1.779	0.736
rs8042532_G	cg05578595	Adiponectin	4.044	9.280	0.663
rs2927324_C	cg05578595	Adiponectin	5.878	31.560	0.852
rs12051272_T	cg05578595	Adiponectin	-1.967	2.146	0.360
rs731839_G	cg05578595	Adiponectin	-0.609	0.770	0.429

Table S3b: Mendelian randomization analysis using SNPs with evidence of association with adiponectin from GWAS as instrumental variables to investigate reverse causation at *ADIPOQ*

SNP	CpG site	Trait	Beta	Standard Error	P-value
rs190934192_A	cg25035485	Apo B	4.202	23.047	0.855
rs629301_G	cg25035485	Apo B	14.004	42.476	0.742
rs1260326_T	cg25035485	Apo B	2.269	2.880	0.431
rs6756629_A	cg25035485	Apo B	1.178	0.676	0.082
rs144064722_G	cg25035485	Apo B	10.567	88.540	0.905
rs182695896_C	cg25035485	Apo B	-3.517	12.705	0.782
rs10056811_A	cg25035485	Apo B	-0.525	4.150	0.899
rs4722043_C	cg25035485	Apo B	0.078	1.094	0.943
rs115849089_A	cg25035485	Apo B	-9.912	64.961	0.879
rs2980875_G	cg25035485	Apo B	-0.420	0.816	0.607
rs635634_T	cg25035485	Apo B	-44.950	890.435	0.960
rs964184_G	cg25035485	Apo B	-1.430	0.936	0.127
rs142130958_A	cg25035485	Apo B	-5.004	19.134	0.794
rs150617279_A	cg25035485	Apo B	0.962	1.203	0.424
rs1081105_C	cg25035485	Apo B	-2.328	4.902	0.635
rs1883711_C	cg25035485	Apo B	0.099	0.674	0.883

Table S3c: Mendelian randomization analysis using SNPs with evidence of association with apolipoprotein B from GWAS as instrumental variables to investigate reverse causation at *APOB*

SNP	CpG site	Trait	Beta	Standard Error	P-value
rs190934192_A	cg00908766	Apo B	-0.343	0.515	0.506
rs1260326_T	cg00908766	Apo B	5.730	16.260	0.725
rs6756629_A	cg00908766	Apo B	3.700	4.976	0.457
rs144064722_G	cg00908766	Apo B	-21.624	355.829	0.952
rs182695896_C	cg00908766	Apo B	-0.658	0.824	0.425
rs10056811_A	cg00908766	Apo B	0.111	0.737	0.881
rs4722043_C	cg00908766	Apo B	-0.057	0.802	0.944
rs115849089_A	cg00908766	Apo B	1.003	0.835	0.230
rs2980875_G	cg00908766	Apo B	0.473	0.831	0.569
rs635634_T	cg00908766	Apo B	22.436	206.885	0.914
rs964184_G	cg00908766	Apo B	-15.376	78.864	0.845
rs142130958_A	cg00908766	Apo B	2.558	4.578	0.577
rs150617279_A	cg00908766	Apo B	4.708	19.372	0.808
rs1081105_C	cg00908766	Apo B	-28.185	594.353	0.962
rs1883711_C	cg00908766	Apo B	0.157	1.056	0.882

Table S3d: Mendelian randomization analysis using SNPs with evidence of association with apolipoprotein B from GWAS as instrumental variables to investigate reverse causation at *SORT1*

SNP	CpG site	Trait	Beta	Standard Error	P-value
rs7553007_A	cg04111102	C-reactive protein	-6.406	7.637	0.402
rs6734238_G	cg04111102	C-reactive protein	-0.473	0.935	0.613
rs7748513_G	cg04111102	C-reactive protein	2.352	3.575	0.511
rs7979473_A	cg04111102	C-reactive protein	-1.538	1.883	0.414
rs1183910_A	cg04111102	C-reactive protein	-11.801	70.720	0.868
rs2259816_T	cg04111102	C-reactive protein	-0.507	1.846	0.784
rs4420638_G	cg04111102	C-reactive protein	-14.441	88.345	0.870

Table S3e: Mendelian randomization analysis using SNPs with evidence of association with C-reactive protein from GWAS as instrumental variables to investigate reverse causation at *LEPR*

SNP	CpG site	Trait	Beta	Standard Error	P-value
rs646776_C	cg19610905	Cholesterol	-8.414	13.767	0.541
rs558971_A	cg19610905	Cholesterol	0.260	0.700	0.710
rs9306897_T	cg19610905	Cholesterol	1.047	1.266	0.409
rs515135_T	cg19610905	Cholesterol	-2.715	2.897	0.349
rs780093_T	cg19610905	Cholesterol	2.606	8.452	0.758
rs6544713_T	cg19610905	Cholesterol	-1.870	2.846	0.511
rs6882076_T	cg19610905	Cholesterol	0.497	1.436	0.730
rs9987289_A	cg19610905	Cholesterol	0.294	5.115	0.954
rs1883025_T	cg19610905	Cholesterol	9.927	41.901	0.813
rs579459_C	cg19610905	Cholesterol	-3.494	4.691	0.457
rs1535_G	cg19610905	Cholesterol	0.197	0.077	0.011
rs10468017_T	cg19610905	Cholesterol	-1.610	2.099	0.443
rs2000999_A	cg19610905	Cholesterol	-2.258	2.097	0.282
rs2156552_A	cg19610905	Cholesterol	1.762	1.360	0.196
rs6511720_T	cg19610905	Cholesterol	-1.389	1.847	0.452
rs2228603_T	cg19610905	Cholesterol	-2.052	2.096	0.328

Table S3f: Mendelian randomization analysis using SNPs with evidence of association with cholesterol from GWAS as instrumental variables to investigate reverse causation at *FADS1*

SNP	CpG site	Trait	Beta	Standard Error	P-value
rs144064722_G	cg04087571	Apo AI	-0.028	2.809	0.992
rs1461729_A	cg04087571	Apo AI	8.999	78.078	0.908
rs75835816_C	cg04087571	Apo AI	-2.291	3.653	0.531
rs1883025_T	cg04087571	Apo AI	15.825	54.031	0.770
rs174594_C	cg04087571	Apo AI	-2.513	2.835	0.376
rs261291_C	cg04087571	Apo AI	1.106	0.840	0.188
rs73424577_G	cg04087571	Apo AI	0.491	0.934	0.599
rs6507939_A	cg04087571	Apo AI	6.219	8.531	0.466

Table S3g: Mendelian randomization analysis using SNPs with evidence of association with apolipoprotein A1 from GWAS as instrumental variables to investigate reverse causation at *APOA1*

SNP	CpG site	Trait	Beta	Standard Error	P-value
rs2228145_C	cg21160290	Interleukin-6	0.013	0.183	0.942

Table S3h: Mendelian randomization analysis using SNPs with evidence of association with interleukin-6 from GWAS as instrumental variables to investigate reverse causation at *ABO*

SNP	CpG site	Trait	Beta	Standard Error	P-value
rs643434_A	cg02856953	Interleukin-6	0.173	0.194	0.372

Table S3i: Mendelian randomization analysis using SNPs with evidence of association with interleukin-6 from GWAS as instrumental variables to investigate reverse causation at *IL6R*

SNP	Gene	CpG	Trait	Sample Size	Beta	SE	P-value
rs266772	*ADIPOQ*	cg05578595	Adiponectin	605	-0.901	0.210	2.01E-05
rs687621	*ABO*	cg21160290	Interleukin-6	605	-2.823	1.940	0.146
rs13375019	*LEPR*	cg04111102	C-reactive protein	605	-0.406	0.142	0.005
rs7549250	*IL6R*	cg02856953	Interleukin-6	605	3.123	3.854	0.418
rs169109	*ADIPOQ*	cg05578595	Adiponectin	605	-0.435	0.169	0.009
rs541041	*APOB*	cg25035485	Apo B	605	0.427	0.165	0.010
rs7528419	*SORT1*	cg00908766	Apo B	605	0.264	0.071	2.04E-04
rs625145	*APOA1*	cg04087571	Apo A1	605	-0.316	0.112	0.005
rs174544	*FADS1*	cg19610905	Cholesterol	605	-0.245	0.109	0.024
rs6749422	*ADCY3*	cg01884057	Body mass index	792	0.144	0.054	0.008

Table S4: Results of Mendelian randomization analysis between DNA methylation (from cord blood) and traits

SNP	Gene	CpG	Trait	Sample Size	Beta	SE	P-value
rs266772	*ADIPOQ*	cg05578595	Adiponectin	647	-0.900	0.190	2.75E-06
rs687621	*ABO*	cg21160290	Interleukin-6	647	-0.278	0.059	2.55E-06
rs13375019	*LEPR*	cg04111102	C-reactive protein	647	-0.394	0.108	2.89E-04
rs7549250	*IL6R*	cg02856953	Interleukin-6	647	0.708	0.319	0.027
rs169109	*ADIPOQ*	cg05578595	Adiponectin	647	-0.445	0.172	0.010
rs541041	*APOB*	cg25035485	Apo B	647	0.245	0.100	0.015
rs7528419	*SORT1*	cg00908766	Apo B	647	0.235	0.058	5.99E-05
rs625145	*APOA1*	cg04087571	Apo A1	647	-0.255	0.076	8.95E-04
rs174544	*FADS1*	cg19610905	Cholesterol	647	-0.248	0.089	0.005
rs6749422	*ADCY3*	cg01884057	Body mass index	846	0.099	0.05	0.043

Table S5: Results of Mendelian randomization analysis between DNA methylation (from blood at age 15.5) and traits

Tables S6

CpG effect			IL6 effect		
**SNP**	**snp_prob**	**snp_log10bf**	**SNP**	**snp_prob**	**snp_log10bf**
rs200533593_A	0.994	3.628	rs200533593_A	0.232	0.892
rs116552240_A	0.569	1.533	rs687621_G	0.154	0.670
rs543040_T	0.312	1.069	rs116552240_A	0.123	0.558
rs644234_G	0.298	1.039	rs687289_A	0.109	0.499
rs493246_A	0.199	0.807	rs657152_A	0.106	0.487
rs8176646_C	0.084	0.376	rs8176646_C	0.058	0.200
rs200700167_A	0.071	0.293	rs544873_A	0.057	0.191
rs676457_T	0.067	0.266	rs582094_T	0.055	0.177
rs612169_G	0.067	0.266	rs582118_G	0.054	0.167
rs491626_T	0.062	0.233	rs494242_T	0.053	0.159

Table S6a: Bivariate fine mapping results at the *ABO* locus

CpG effect			BMI effect		
**SNP**	**snp_prob**	**snp_log10bf**	**SNP**	**snp_prob**	**snp_log10bf**
rs6737082_C	0.886	2.595	rs6737082_C	0.067	0.561
rs59086897_A	0.815	2.346	rs6746013_G	0.062	0.526
rs10865321_C	0.521	1.740	rs10182181_G	0.062	0.520
rs10865322_G	0.479	1.667	rs10182458_G	0.060	0.512
rs6746013_G	0.175	1.030	rs6752378_A	0.058	0.493
rs58048722_C	0.099	0.742	rs6749422_G	0.053	0.452
rs2118826_A	0.092	0.709	rs59086897_A	0.050	0.422
rs1470039_G	0.090	0.701	rs10203482_C	0.035	0.268
rs4665736_C	0.088	0.690	rs10185143_C	0.033	0.239
rs1172294_G	0.081	0.650	rs58048722_C	0.031	0.213

Table S6b: Bivariate fine mapping results at the *ADCY3* locus

CpG effect			Adiponectin effect		
**SNP**	**snp_prob**	**snp_log10bf**	**SNP**	**snp_prob**	**snp_log10bf**
rs266772_T	0.367	0.732	rs115527175_T	0.821	1.631
rs150411458_C	0.192	0.346	rs74577862_A	0.126	0.129
rs78800820_C	0.156	0.234	rs266772_T	0.124	0.12
rs146920076_A	0.156	0.234	rs201071850_G	0.081	-0.088
rs201071850_G	0.140	0.182	rs150411458_C	0.072	-0.142
rs74577862_A	0.126	0.128	rs78800820_C	0.054	-0.275
rs76786086_T	0.123	0.115	rs146920076_A	0.054	-0.275
rs141134215_A	0.120	0.106	rs76786086_T	0.029	-0.559
rs189214085_G	0.120	0.106	rs202214354_G	0.027	-0.580
rs143257534_T	0.120	0.106	rs1011551_T	0.026	-0.608

Table S6c: Bivariate fine mapping results at the *ADIPOQ* (low freq) locus

CpG effect			Adiponectin effect		
**SNP**	**snp_prob**	**snp_log10bf**	**SNP**	**snp_prob**	**snp_log10bf**
rs169109_G	0.472	1.001	rs169109_G	0.421	0.911
rs67654560_A	0.374	0.826	rs34587333_A	0.306	0.695
rs34587333_A	0.294	0.669	rs266728_G	0.282	0.645
rs266728_G	0.279	0.638	rs58575091_T	0.161	0.333
rs58575091_T	0.266	0.609	rs864264_C	0.043	-0.297
rs266720_A	0.183	0.400	rs67654560_A	0.034	-0.399
rs864264_C	0.167	0.353	rs266750_C	0.027	-0.502
rs843991_T	0.161	0.333	rs266759_T	0.026	-0.528
rs16861153_A	0.111	0.145	rs201648331_T	0.026	-0.531
rs266754_T	0.085	0.018	rs843991_T	0.025	-0.541

Table S6d: Bivariate fine mapping results at the *ADIPOQ* (common) locus

CpG effect			APOAI effect		
**SNP**	**snp_prob**	**snp_log10bf**	**SNP**	**snp_prob**	**snp_log10bf**
rs688456_T	0.957	2.519	rs625145_T	0.225	0.639
rs12225230_C	0.494	1.166	rs688456_T	0.167	0.478
rs11216162_A	0.335	0.879	rs11216162_A	0.059	-0.028
rs61903423_A	0.153	0.435	rs12225230_C	0.053	-0.078
rs61905689_T	0.105	0.244	rs7932655_A	0.052	-0.082
rs7928320_T	0.077	0.095	rs34999185_A	0.051	-0.098
rs59511712_T	0.070	0.054	rs61905717_A	0.050	-0.103
rs17120244_T	0.069	0.049	rs7928320_T	0.049	-0.110
rs7946729_T	0.063	0.049	rs17120244_T	0.047	-0.127
rs7932655_A	0.059	-0.027	rs7930783_A	0.044	-0.166

Table S6e: Bivariate fine mapping results at the *APOA1* locus

CpG effect			APOB effect		
**SNP**	**snp_prob**	**snp_log10bf**	**SNP**	**snp_prob**	**snp_log10bf**
rs541041_G	0.148	1.285	rs580889_C	0.108	1.128
rs581411_G	0.127	1.211	rs201027918_C	0.100	1.091
rs562338_A	0.122	1.188	rs10692845_T	0.095	1.068
rs515135_T	0.111	1.144	rs548145_T	0.080	0.984
rs563290_G	0.104	1.111	rs541041_G	0.073	0.942
rs10692845_T	0.100	1.093	rs515135_T	0.072	0.939
rs668948_G	0.100	1.091	rs562338_A	0.067	0.901
rs580889_C	0.098	1.080	rs563290_G	0.062	0.865
rs548145_T	0.075	0.958	rs668948_G	0.060	0.855
rs1652416_A	0.016	0.250	rs581411_G	0.060	0.848

Table S6f: Bivariate fine mapping results at the *APOB* locus

CpG effect			APOB effect		
**SNP**	**snp_prob**	**snp_log10bf**	**SNP**	**snp_prob**	**snp_log10bf**
rs646776_C	0.771	1.316	rs4970836_G	0.164	0.080
rs7528419_G	0.207	0.206	rs629301_G	0.159	0.066
rs629301_G	0.020	-0.897	rs599839_G	0.123	-0.064
rs12740374_T	0.002	-1.924	rs646776_C	0.116	-0.092
rs4970836_G	0	-6.704	rs7528419_G	0.108	-0.130
rs583104_G	0	-7.790	rs583104_G	0.107	-0.132
rs1277930_G	0	-9.292	rs1277930_G	0.080	-0.272
rs599839_G	0	-9.556	rs12740374_T	0.055	-0.446
rs660240_T	0	-9.783	rs602633_T	0.033	-0.676
rs57677983_C	0	-9.783	rs57677983_C	0.029	-0.743

Table S6g: Bivariate fine mapping results at the *SORT1* locus

CpG effect			Cholesterol effect		
**SNP**	**snp_prob**	**snp_log10bf**	**SNP**	**snp_prob**	**snp_log10bf**
rs174559_A	0.986	3.274	rs174548_G	0.134	0.612
rs1535_G	0.527	1.469	rs174560_C	0.116	0.540
rs5792235_C	0.226	0.887	rs174555_C	0.106	0.494
rs174561_C	0.221	0.875	rs199977718_C	0.102	0.479
rs174555_C	0.206	0.836	rs174549_A	0.102	0.477
rs174556_T	0.197	0.812	rs174561_C	0.079	0.356
rs174544_A	0.179	0.760	rs174556_T	0.076	0.338
rs174549_A	0.154	0.682	rs28456_G	0.043	0.076
rs174568_T	0.039	0.031	rs174544_A	0.042	0.059
rs174537_T	0.038	0.014	rs174574_A	0.035	-0.020

Table S6h: Bivariate fine mapping results at the *FADS1* locus

CpG effect			IL6 effect		
**SNP**	**snp_prob**	**snp_log10bf**	**SNP**	**snp_prob**	**snp_log10bf**
rs10908837_G	0.187	0.607	rs12118721_T	0.130	0.420
rs4845618_G	0.164	0.538	rs10908837_G	0.124	0.397
rs6687726_A	0.163	0.535	rs6687726_A	0.113	0.349
rs12117832_A	0.150	0.490	rs12117832_A	0.079	0.179
rs7536152_A	0.141	0.459	rs4845618_G	0.076	0.161
rs12118721_T	0.136	0.440	rs10908836_C	0.056	0.020
rs12129500_T	0.126	0.403	rs4553185_C	0.047	-0.059
rs6686750_A	0.125	0.400	rs6694817_T	0.043	-0.102
rs6689393_A	0.124	0.394	rs10908838_T	0.042	-0.111
rs7526131_G	0.123	0.393	rs34926346_A	0.039	-0.143

Table S6i: Bivariate fine mapping results at the *IL6R* locus

CpG effect			IL6 effect		
**SNP**	**snp_prob**	**snp_log10bf**	**SNP**	**snp_prob**	**snp_log10bf**
rs6693842_C	0.754	2.232	rs6700896_T	0.122	0.887
rs4655764_T	0.583	1.890	rs200641814_TA	0.078	0.670
rs59508186_A	0.353	1.481	rs7535218_A	0.067	0.602
rs4655582_G	0.173	1.066	rs7524581_T	0.051	0.476
rs10889574_A	0.141	0.959	rs10443261_C	0.039	0.349
rs11208715_T	0.139	0.951	rs7518710_G	0.037	0.328
rs79653480_A	0.139	0.951	rs7541434_A	0.034	0.289
rs12021623_C	0.139	0.951	rs12067936_A	0.032	0.267
rs78052673_C	0.139	0.951	rs7515766_G	0.030	0.241
rs6664374_T	0.129	0.916	rs12753193_G	0.029	0.218

Table S6j: Bivariate fine mapping results at the *LEPR* locus

SNP	Gene	CpG	Pr(single var | trait)	Concordance rate	P_{JLIM}
rs266772	*ADIPOQ*	cg05578595	0.746	0.188	$<10^{-6}$
rs200533593	*ABO*	cg21160290	0.699	0.024	NA
rs6693842	*LEPR*	cg04111102	0.785	0.213	0.95
rs10908837	*IL6R*	cg02856953	0.929	0.069	NA
rs541041	*APOB*	cg25035485	0.736	0.028	1
rs169109	*ADIPOQ*	cg05578595	0.725	0.048	$<10^{-6}$
rs646776	*SORT1*	cg00908766	0.984	0.360	1
rs688456	*APOA1*	cg04087571	0.841	0.077	1
rs174559	*FADS1*	cg19610905	0.809	0.488	0.99
rs6737082	*ADCY3*	cg01884057	0.761	0.012	$<10^{-6}$

Table S7: Results of bivariate fine mapping concordance and joint likelihood mapping (JLIM) SNP – Single Nucleotide Polymorphism, Gene – likely implicated gene, CpG – 450K probe ID, Pr(single var | trait) – the posterior probability that a single causal variant is responsible for the observed effect on complex traits (based on a maximum of 5 causal variants), Concordance rate – concordance rate generated from bivariate fine mapping results, P_{JLIM} – P value reported by the JLIM method

mQTL	Gene	CpG	CpG effect	hQTL	Histone Mark	hQTL effect	hQTL P	2SMR	P-value
rs625145	*APOA1*	cg04087571	-0.884 (0.044)	rs688456	H3K4ME3	0.913 (0.166)	5.58×10^{-7}	-1.033 (0.195)	1.13×10^{-7}
rs625145	*APOA1*	cg04087571	-0.884 (0.044)	rs688456	H3K27AC	0.758 (0.176)	4.95×10^{-5}	-0.857 (0.204)	2.54×10^{-5}
rs625145	*APOA1*	cg04087571	-0.884 (0.044)	rs688456	H3K4ME1	0.930 (0.165)	3.04×10^{-7}	-1.052 (0.194)	5.74×10^{-8}
rs10908837	*IL6R*	cg02856953	-0.303 (0.039)	rs59632925	H3K4ME1	0.620 (0.138)	2.53×10^{-5}	-2.045 (0.526)	1.01×10^{-4}
rs10908837	*IL6R*	cg02856953	-0.303 (0.039)	rs59632925	H3K27AC	0.658 (0.124)	1.14×10^{-6}	-2.172 (0.496)	1.18×10^{-5}

Table S8: Results of analysis investigating causal relationship between methylation and histone modification using Two-Sample Mendelian randomization mQTL – methylation quantitative trait loci, Gene – likely implicated gene, CpG – 450K probe ID, CpG effect – effect estimate of SNP on methylation, hQTL – histone quantitative trait loci,, hQTL effect - effect estimate of LD SNP on histone mark from Grubert et al (2015), hQTL P – P-value for hQTL effect, 2SMR effect – effect estimates from 2-Sample MR analysis, P-value – P-value for observed 2SMR effect
